# Route of Vaccine Administration Influences the Impact of Fms-Like Tyrosine Kinase 3 Ligand (Flt3L) on Chlamydial-Specific Protective Immune Responses

**DOI:** 10.3389/fimmu.2019.01577

**Published:** 2019-07-04

**Authors:** Roshan Pais, Yusuf Omosun, Joseph U. Igietseme, Kohtaro Fujihashi, Francis O. Eko

**Affiliations:** ^1^Department of Microbiology, Biochemistry and Immunology, Morehouse School of Medicine, Atlanta, GA, United States; ^2^Molecular Pathogenesis Laboratory, Centers for Disease Control and Prevention, Atlanta, GA, United States; ^3^Department of Pediatric Dentistry, Institute of Oral Health Research, University of Alabama at Birmingham, Birmingham, AL, United States

**Keywords:** *Chlamydia trachomatis*, *C. muridarum*, PmpD, PorB, Flt3L, vaccine delivery route, protective immunity

## Abstract

We tested the hypothesis that the impact of the Fms-like tyrosine kinase 3-ligand (Flt3L; FL) on recombinant *Vibrio cholerae* ghost (rVCG) vaccine-induced chlamydial immunity is influenced by route of vaccine delivery. Female C57BL/6J mice were immunized rectally (IR) or intramuscularly (IM) with rVCG co-expressing the *Chlamydia trachomatis* PmpD and PorB proteins (rVCG- PmpD/PorB) with and without FL or glycoprotein D of HSV-2 (rVCG-gD2) as antigen control. Vaccine evaluation was based on measurement of T cell proliferation, Th1/Th2 cytokine, and humoral responses at systemic and mucosal compartments, and protection against intravaginal challenge infection. Results revealed that high levels of CD4+ T cell-mediated and humoral immune responses, were elicited in mice as a function of both IR and IM immunization. Unexpectedly, co-administration of vaccine with FL enhanced specific Th1-type cytokine levels and T cell proliferative responses following IR but not IM immunization. While administration of vaccine with FL enhanced the specific mucosal and systemic IgA antibody responses following both immunization routes, IgG2c responses were not enhanced following IR delivery. The vaccine-induced immune effectors protected mice against live heterologous *C. muridarum* infection irrespective of route of vaccine administration, with the regimen incorporating FL having a protective advantage. Further evaluation showed that protection afforded by the FL adjuvanted vaccine was facilitated by CD4+ T cells, as indicated by reduction in the intensity and duration of genital chlamydial shedding by naïve mice following adoptive transfer of immune CD4+ T cells. Taken together, the results indicate that comparable protective immunity, which is enhanced by co-delivery with FL, is elicited in the female genital tract against *Chlamydia* infection after mucosal and systemic administration, highlighting the ability of FL to function as an effective immunostimulator at both mucosal and systemic sites. The differential modulation of humoral and cellular immune responses, and protective immunity afforded by the FL adjuvanted vaccine following IR administration indicates that the immunomodulatory impact of FL on chlamydial-specific immunity is influenced by the route of vaccine administration. Thus, targeting of VCG-based vaccines to antigen presenting cells by co-delivery with FL is a feasible immunization approach for inducing effective chlamydial immunity in the female genital tract.

## Introduction

*Chlamydia trachomatis* remains the commonest cause of sexually transmitted diseases (STDs) of bacterial etiology worldwide and can lead to severe irreversible complications in women in the absence of appropriate therapeutic intervention. Such complications include, pelvic inflammatory disease, ectopic pregnancy, and barrenness (1, 2). Management of these infections in the USA alone results in an annual expenditure in excess of $2 billion (3). Currently, azithromycin or doxycycline are first-line recommendations for treatment of non-pregnant patients with urogenital or oropharyngeal *Chlamydia* (4). However, since most infections are asymptomatic, many infected individuals are not treated, suggesting that the development and use of an effective vaccine may be the best approach for preventing these infections and the associated sequelae worldwide (5–7). Despite concerted efforts, there is currently no licensed *Chlamydia* vaccine. Because the use of live or inactivated whole cell chlamydial vaccines led to development of immunopathology in vaccines (8), current vaccine efforts have focused on use of chlamydial subunit antigens. In addition to the chlamydial outer membrane protein, MOMP (9–11), a number of immunogenic proteins have been predicted as possible vaccine candidates (12–14). Results from several laboratories indicate that single component vaccines only induce partial protection (3, 15, 16), suggesting that multicomponent vaccines combined with effective delivery may lead to more efficacious chlamydial vaccines.

The inability of current *Chlamydia* vaccine candidates to induce sterilizing immunity may be related to the use of inadequate delivery systems or inability to stimulate an appropriate immune response. Thus, the use of an effective delivery system in combination with an appropriate adjuvant may lead to the induction of superior protective immunity. In particular, a delivery system capable of simultaneously presenting more than one antigen may constitute a viable *Chlamydia* vaccination strategy. In this regard, rVCG delivery system has been shown to be effective in delivering multiple antigens concurrently to the immune system (16–18). We previously showed that a recombinant *Vibrio cholerae* ghost (rVCG)-based multisubunit vaccine composed of the *C. trachomatis* polymorphic outer membrane protein D (PmpD) and porin B (PorB) proteins (8, 19, 20) induced long-term, cross protective immune responses following intramuscular (IM), and rectal (IR) immunization in mice (17, 18, 21). These studies established that IM and IR immunization were viable systemic and mucosal routes for inducing female genital tract immunity. However, the influence of incorporating immunomodulators in the VCG platform on the magnitude and quality of chlamydial-specific protective immune responses following rectal mucosal and intramuscular systemic vaccine delivery has not previously been investigated.

In addition to a multisubunit design approach and utility of an efficient delivery system, vaccines based on subunit components often necessitate the inclusion of immune modulators (adjuvants) to augment protective immunity. A number of delivery systems and adjuvants have been used to augment protective immune responses to chlamydial vaccine antigens (22–26). The Fms-like tyrosine kinase 3 ligand (Flt3L) is a four helical bundle cytokine and growth factor that is structurally homologous to stem cell factor (SCF) and colony stimulating factor 1 (CSF-1) (27). It binds to the fms-like tyrosine kinase receptor Flt3/Flk2 (CD135) and in collaboration with other growth factors, stimulates the proliferation and differentiation of various blood cell progenitors. For example, it dramatically increases immune cell number and has been exploited for dendritic cell (DC) expansion (28). Intranasal administration of Flt3L with P6 protein of non-typeable *Haemophilus influenzae* (NTHi) to mice increased dendritic cell number in the nasal-associated lymphoid tissue, significantly elevated P6-specific nasal mucosal IgA and serum IgG titers and boosted nasopharyngeal NTHi clearance (29). A combination of Flt3L and CpG oligodeoxynucleotide adjuvants elicited secretory-IgA immune responses that protected aged mice against *Streptococcus pneumoniae* infection (30). A mixture of Flt3L and Granulocyte macrophage-colony stimulating factor (GM-CSF) considerably enhanced the maturation of splenic DC and their ability to present antigen to immune T cells (28) and elicited mucosal immunity to influenza in aging (31).

On the basis of the ability of Flt3L to expand DCs and enhance protective immunity following immunization, in the present study we sought to evaluate the impact of Flt3L (FL) on chlamydial-specific protective immune responses following rectal mucosal and intramuscular systemic vaccine delivery. Our results showed that FL enhanced the antigen-specific Th1 cytokine responses following IR but not IM immunization and the mucosal and systemic IgA and IgG2c antibody responses following IM immunization. IgG2c responses were not enhanced by FL following IR mucosal delivery. The vaccine-induced immune effectors protected mice against live heterologous *C. muridarum* infection irrespective of route of vaccine administration, with the regimen incorporating FL having a protective advantage. Further evaluation showed that the protection was mediated by CD4+ T cells, as indicated by reduction in the intensity of genital chlamydial shedding by naïve mice following adoptive transfer of immune CD4+ T cells. These results suggest that comparable protective immunity can be stimulated in the female genital tract against *Chlamydia* infection subsequent to mucosal and systemic vaccination, highlighting the ability of FL to function as an effective immunostimulator at both mucosal and systemic sites.

## Materials and Methods

### Ethics Statement

This study was carried out in strict accordance with the recommendations in the Guide for the Care and Use of Laboratory Animals of the National Institutes of Health. The Institutional Animal Care and Use Committee (IACUC) of Morehouse School of Medicine (MSM) (Assurance number A3381-01) approved the study protocol (Protocol Number: 16-15). Six-week-old female C57BL/6J mice (stock number 000664) (The Jackson Laboratory, Bar Harbor, ME) were used in this study. Mice were allowed to acclimate for 10 days in the MSM Center for Laboratory Animal Resources (CLAR) facility prior to experimentation. All immunizations, challenge and surgery were performed under ketamine/xylazine anesthesia, and conscious efforts were made to minimize suffering.

### *Chlamydia* Stocks and Antigens

The *C. trachomatis* serovar D and *C. muridarum* organisms used in the present study were titrated on HeLa cell monolayers and chlamydial elementary bodies (EBs) were purified by renografin density gradient centrifugation and stored at −70°C. Chlamydial antigen was prepared by exposing purified EBs to UV radiation for 3 h and prepared antigen was kept at −70°C until used.

### Construction of Vaccine Antigens

**Figure 1:**
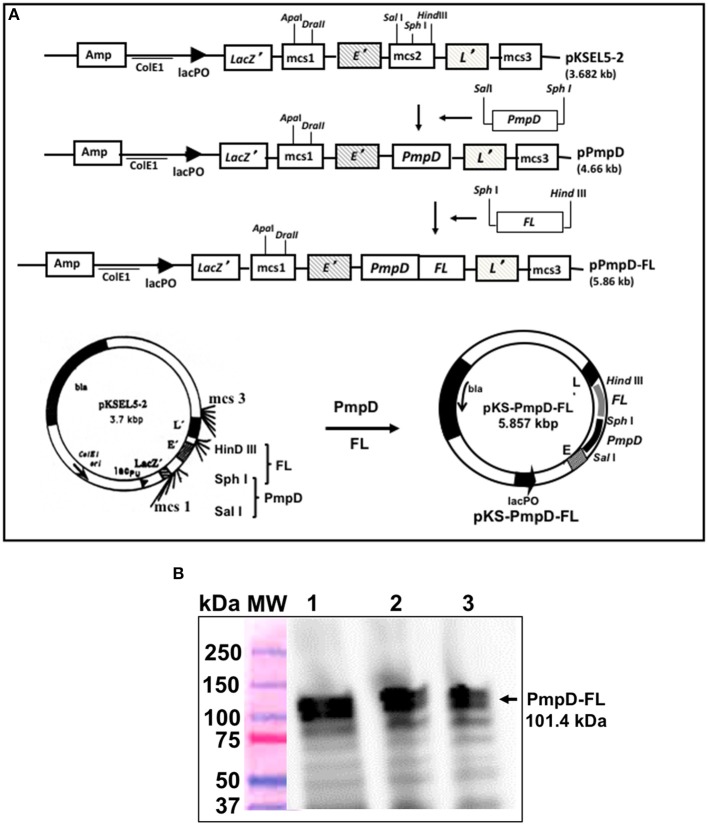
Construction of the inner membrane targeting plasmid pKS-PmpD/FL and expression of PmpD-FL fusion proteins. **(A)** Plasmid pKS-PmpD/FL was generated by sequentially inserting the N-terminal (600 amino-acid) segment of the *C. trachomatis* (serovar D) *PmpD* gene and the *flt3L* gene encoding the full-length mouse Flt3L (FL) in frame with the membrane spanning domains of genes E' and L' of phages PhiX174 and MS2, respectively contained in the plasmid vector pKSEL-5-2. **(B)** Protein expression was detected by Western immunoblotting analysis using mAb to mouse FL. Lanes 1, 2, and 3, PmpD/FL expressed from plasmid pKS-PmpD/FL at 4, 2, and 1 h, respectively. MW, Molecular weight marker in kilodaltons (kDa).

The PmpD gene was obtained from *C. trachomatis* serovar D genomic DNA by PCR amplification using the following primers: (PmpD-*Sal* 1 Forward 5′- <UNDACT TGAERLINE> gct gac TCT GTA GTA GCA GCT−3′ and PmpD-*Sph* 1 Reverse 5′- <UNDGAT TATERLINE> gca tgc CAA ACT AGC AAT ATT-3′) incorporating the *Sal* I and *Sph* I restriction sites. Construction of the vaccine vector pKS-PmpD harboring the PmpD coding sequence was accomplished by inserting the amplified *PmpD* gene into the membrane-targeting vector, pKSEL5-2 at the C-terminal region of gene *E'* under the transcriptional control of the *lacPO* promoter to yield pKS-PmpD. Also, the mouse Flt3L (FL) cDNA sequence was amplified from plasmid pORF9-mFlt3L (InvivoGen, San Diego, CA) by PCR using the primers: (FL-*Sph* I Forward 5′- <UNDEGAACGARLINE>tctagaACACCTGA-CTGTTAC-3′ and FL-*Hind* III Reverse 5′- <UNDETCTAATRLINE>gctgacGGG-ATGGGAGGGGAG-3′ incorporating the *Sph* I and *Hind* III restriction sites. The vaccine vector harboring the PmpD and FL coding sequences was then constructed by introducing the amplified *Flt3l* gene into plasmid, pKS-PmpD to generate plasmid pKS-PmpD/FL (Figure 1A). Construction of the periplasmic targeting vector pMAL-PorB harboring the full-length PorB gene without its signal peptide and expression of the 80-kDa recombinant PorB fusion (PorB-MBP) protein has previously been described (18).

### Expression of PmpD/FL by Immunoblotting Analysis

Plasmid pKS-PmpD/FL was transformed into *V. cholerae* O1 strain V912 by electroporation and expression of the PmpD/FL fusion protein was evaluated by immunoblotting analysis as previously described (3). Briefly, a culture of V912 (pKS-PmpD/FL) was grown to mid-log phase at 37°C, and rPmpD/FL expression was induced by the addition of isopropyl-β-D-thiogalactopyranoside (IPTG; Roche Diagnostics, Indianapolis, IN) to a final concentration of 2 mM; the culture was then incubated for a further 60 min. At the indicated times points, samples were removed and solubilized in sample buffer containing 2-ME (Invitrogen Life Technologies, Carlsbad, CA), and separated by SDS-PAGE. Following protein transfer, rPmpD/FL was detected using mAb to mouse FL (Catalog # AF427-SP; R&D Systems, Minneapolis, MN).

### Production of rVCG Vaccines

*V. cholerae* O1 strain H1 harboring pPmpD or pPmpD/FL or pMAL-PorB and the lysis plasmid pDKLO1 was cultured in brain heart infusion (BHI) broth at 37°C and the various rVCG vaccines were produced by genetic inactivation of the *V. cholerae* cells as previously described (3, 32). This essentially involves introduction by protein E of a lysis tunnel structure through the cell envelope complex, which leads to expulsion of the entire cytoplasmic material from the cells, resulting in empty cell envelopes. The cell envelopes (VCG) were then harvested by centrifugation, washed several times with a low ionic buffer, freeze-dried and stored refrigerated until used.

### Immunization, Challenge, and Analysis of Protective Immunity

The vaccine candidates consisted of recombinant VCG expressing the chlamydial porin B (PorB) protein or polymorphic membrane protein D protein (PmpD) or PmpD fused to mouse Flt3L (PmpD-FL). Recombinant VCG expressing glycoprotein D from HSV-2, which is a chlamydial irrelevant antigen (rVCG-gD2) was used as a vaccine antigen control. Groups of female mice were immunized IR (16/group) or IM (12/group) with 50 μl PBS containing 500 μg each of lyophilized rVCG-PorB and rVCG-PmpD or rVCG-PmpD/FL or rVCG-gD2 (1 mg) or FL (150 ng) control on days 0, 14, and 28 as previously described (16). A mock immunized group received 50 μl of PBS alone. Mice were sedated using a combination of ketamine (75 mg/kg Ketaset, Zoetis, Florham Park, NJ) and xylazine (15 mg/kg Anased, Lloyd, Shenandoah, IA) before each immunization. Mice (8/group; IR and 6/group; IM) were challenged intravaginally 2 weeks after the last immunization with 5 × 10^4^ IFUs of live *C. muridarum* in 5 μl of SPG buffer. Seven days before challenge, each mouse received 2.5 mg medroxyprogesterone (Depo Provera; Pharmacia UpJohn Co., Kalamazoo, MI) by subcutaneous injection to harmonize the estrous cycle and facilitate a productive infection. After challenge, mice were monitored daily to ascertain their health status. Cervicovaginal swabs obtained every 7 days were cultured in McCoy cells and vaginal chlamydial burden was assessed by enumerating the number of IFU detected by indirect immunofluorescence using the Pathfinder *Chlamydia* Culture Confirmation System #30701 (BioRad, Hercules, CA) (33). The Log_10_ number of IFU/ml recovered from each mouse was then calculated.

### Assessment of Antigen-Specific Cellular Immune Responses

Immune T cells were isolated from the spleens (SPL) of immunized and control mice (6–8/group) 2weeks after the final booster immunization using the gentleMACS Dissociator. Purification of CD4+ T cells was further accomplished by subjecting cells to positive selection using the Midi magnetic bead-activated cell sorting (MidiMACS) purification system in combination with CD4-specific MACS microbeads (Miltenyi Biotech, Auburn, CA). CD4+ T cell purity was determined by flow cytometry to be > 95% using an APC-conjugated anti-CD4 monoclonal antibody (Pharmingen, San Diego, CA). Splenocytes isolated from naive mice were treated with mitomycin C (25 μg/10^7^ cells) for 20 min and used as a source of antigen-presenting cells (APCs). The amount of *Chlamydia*-specific Th1/Th2 cytokines secreted by splenic immune CD4+ T cells was assessed as previously described (17). In brief, purified splenic immune CD4^+^ T cells (2 × 10^5^ cells/well) were cultured in triplicates with APCs (2 × 10^5^) and 10 μg/ml of chlamydial antigen in 96-well tissue culture plates. Cultures containing APCs and T cells but without chlamydial antigen were included as controls. After 5 days of culture, harvested supernatants were then assayed for cytokine concentration using a combination of the Bio-Plex cytokine assay kit (IFN-γ, IL-2, TNF-α, IL-10, IL-4) that incorporated cytokine standards and the Bio-Plex Manager software (Bio-Rad, Hercules, CA). The concentration of the different cytokines in each sample was extrapolated from the simultaneously generated standard calibration curve. Data were calculated as the mean values (±S.D.) for triplicate wells for each experiment. The assay was repeated thrice.

### Measurement of T Cell Proliferation

The ability of immune CD4^+^ T cells purified from the spleens of immunized mice to proliferate in response to *in vitro* restimulation with chlamydial antigen in culture was assessed using the 5-Bromo-2′-deoxy-uridine (BrdU) cell proliferation assay kit according to the manufacturer's instructions (Roche Molecular Biochemicals, Indianapolis, IN). Thus, CD4^+^ T cells (10^6^ cells/ml) resuspended in RPMI 1640 medium (Sigma-Aldrich, St. Louis, MO) supplemented with 10% heat-inactivated FBS were cultured with γ-irradiated (3,000 rads) normal mouse spleen cells (10^6^/ml) in the presence or absence of chlamydial antigen (10 μg/ml) at 37°C in 5% CO_2_. After 5 days of culture, the cells were transferred to round-bottom 96-well plates (Corning Glass Work, Acton, MA) and 10 μl of BrdU labeling solution at a final concentration of 10 μM/ml was added to each well and the plates were incubated at 37°C in 5% CO_2_ for 18 h. After fixing, 100 μl of nuclease was added to the cells in each well and incubated at 37°C for 30 min. Following washing, peroxidase-coupled anti-BrdU antibody was added to the cells, incubated for 1 h at 37°C and developed with 2,2'-azino-bis (3-ethylbenzthiazoline-6-sulfonic acid) (ABTS) substrate (100 μl) for 30 min. BrdU incorporation was detected using a scanning multi-well spectrophotometer (Spectra-Max 250 ELISA reader, Molecular Devices, Sunnyvale, CA) and the optical density was read at 405 nm. T cell cultures that excluded chlamydial antigen (non-stimulated cells) were included and served as controls. The stimulation index (SI), defined as the ratio of the absorbance values of stimulated and non-stimulated cells was then calculated.

### PorB Peptide Antibody ELISA

Serum samples of immunized and control mice collected via the retro-orbital plexus 2 weeks after the last boost were assayed for the presence of IgA and IgG2c antibodies to a 15-amino acid synthetic peptide (Syd Labs, Malden, MA) derived from a conserved epitope of *C. trachomatis* PorB (PorB15). Briefly, PorB15 peptide (2.5 μg/well) and 2-fold serially diluted (0.0, 12.5, 25, 50. 100, 250, 500, and 1,000 ng/ml) IgA and IgG2c standards (Southern Biotech, Birmingham, AL) in carbonate buffer (14.2 mM Na_2_CO_3_, 34.9 mM NaHCO_3_, 3.1 mM NaN_3_, pH 9.5) were used to coat the wells of a microliter plate overnight at 4°C and generate a standard calibration curve. After washing, PBS containing 3% bovine serum albumin (BSA) was added to the wells of plates to block non-specific binding and samples of serum (100 μl) diluted 1:10 or vaginal wash (50 μl) diluted 1:2 were then added. After 2 h at 37°C, 100 μl of horseradish peroxidase-conjugated goat anti-mouse IgA (1:500 dilution) or IgG2c (1: 2,000 dilution) (Southern Biotechnology Associates, Inc., Birmingham, Ala.) was added to each well and incubated at 37°C for 1 h. The plates were developed by incubating wells with 50 μl of peroxidase substrate, tetramethylene benzidine (TMB) in the dark for 15 min, the reaction stopped by adding 50 μl of 0.5 M H_2_SO_4_ and the plates were then read on a Microplate reader at 450 nm. Data sets corresponding to absorbance values were generated simultaneously with the standard curve and displayed as mean concentrations (ng/ml) ± SD, representing the mean values from triplicate experiments.

### Role of CD4+ T Cells in Protection Mediated by VCG-Based Chlamydial Vaccine

Mice (8/group) were rectally immunized and boosted twice, 2 weeks apart with rVCG-PmpD/PorB-FL vaccine or controls (PBS, rVCG-gD2 and FL). Two weeks after the last booster dose, splenic immune CD4^+^ T cells (2.5 × 10^7^ cells/mouse; 2.5 spleen equivalents) were adoptively transferred intraperitoneally into naive mice 24 h before intravaginal challenge with live *C. muridarum* (5 × 10^4^ IFUs) in 5 μl of SPG buffer. Vaginal swabs were obtained from individual animals every 3 days to monitor infections and chlamydial burden was assessed as described above.

### Statistical Analysis

The GraphPad Prism 5.0 software (GraphPad Software, Inc., La Jolla, CA) was used to perform statistical analysis on a MAC computer. Analysis of variance (ANOVA) was used for comparing more than two groups while the unpaired Student *t*-test was used for comparing differences between 2 groups. Statistical significance was determined at probability (*p*) values ≤ 0.05.

## Results

### The Generated Vaccine Antigen, E-PmpD/FL-L Was Successfully Expressed

The constructed vaccine vector, pKS-PmpD/FL had the PmpD and FL coding sequences cloned between the C-terminal E' and N-terminal-L' anchors of the membrane-targeting vector, pKSEL5-2 (Figure 1A) such that the PmpD and FL proteins are expressed as an *E*' and *L*' fusion protein (E-PmpD/FL-L). The integrity of the pKS-PmpD/FL plasmid construct was confirmed by gene sequencing. Expression of the 114-kDa recombinant fusion protein (E-PmpD/FL-L) in *V. cholerae* transformed with plasmid pKS-PmpD/FL was detected by immunoblotting analysis using polyclonal Ab to mouse Flt3L (AA_27−189_) (Antibodies-Online, Atlanta, GA) (Figure 1B).

### Rectal and IM Immunization With PmpD/PorB-FL Induced Antigen-Specific Th1 Cytokine Responses

**Figure 2:**
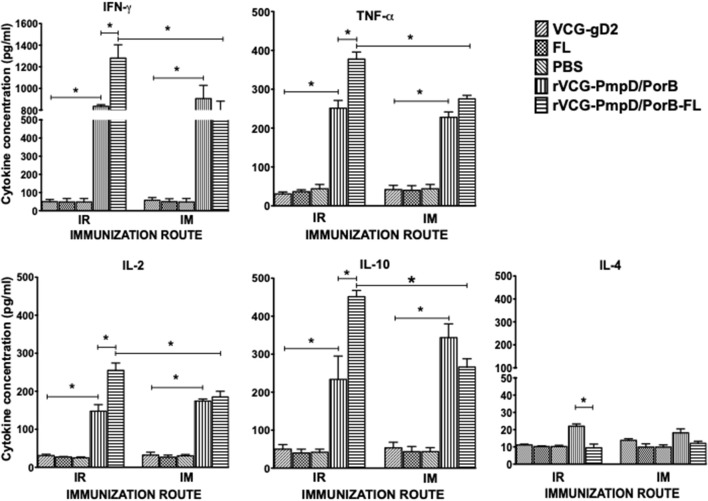
*Chlamydia*-specific Th1/Th2 cytokine responses. Groups of mice were immunized and boosted twice via the IR and IM routes as described in the materials and methods section. Two weeks post-immunization, CD4+ T cells were purified from the spleens of immunized mice and controls and restimulated *in vitro* with chlamydial antigen. The amount of systemic Th1 (IFN-γ, IL-2, and TNF-α), Th2 (IL-4) and IL-10 cytokines contained in supernatants of culture-stimulated cells was measured using Bio-Plex cytokine assay kit in combination with the Bio-Plex Manager software. The concentration of the cytokines in each sample was obtained by extrapolation from a standard calibration curve generated simultaneously. Data were calculated as the mean values (±S.D.) for triplicate cultures for each experiment. The cultures without antigen did not contain detectable levels of cytokine and so the data were excluded from the results. Significant differences between Th1 and Th2 cytokines (IFN-γ and IL-4) were evaluated at (*p < 0.05).

The antigen-specific cell-mediated immune responses induced following immunization were examined by evaluating the magnitude of Th1/Th2 cytokines secreted by purified splenic immune CD4+ T cells upon restimulation with chlamydial antigen 2 weeks post-immunization. The results showed both routes of immunization with rVCG-PmpD/PorB stimulated the production of high levels of antigen-specific Th1-type cytokines as indicated by high concentrations of IFN-γ, IL-2, and TNF-α and low levels of the Th2 cytokine, IL-4 compared to controls (Figure 2). The amount of Th1 cytokines secreted by immune T cells from vaccine-immunized mice was significantly higher (*p* < 0.05) compared to PBS control, irrespective of route of vaccine delivery. Expectedly, T cells from mice immunized with rVCG-gD2 or FL did not secrete appreciable levels of Th1 cytokines. Notably, although Th1 cytokine levels were comparable following rectal mucosal and IM systemic immunization, co-delivery of vaccine with FL significantly enhanced Th1 cytokine levels after IR but not IM immunization. Also, the amounts of Th1 cytokines induced by the FL adjuvanted vaccine was significantly higher (*p* < 0.05) in rectally immunized mice compared to mice immunized by the IM route. Furthermore, although both routes of immunization stimulated the secretion of high levels of the anti-inflammatory cytokine, IL-10, co-delivery of vaccine with FL significantly enhanced IL-10 levels after IR but not IM immunization.

### Immune CD4+ T Cells From IR and IM Immunized Mice Proliferated in Response to Restimulation With Chlamydial Antigen

**Figure 3:**
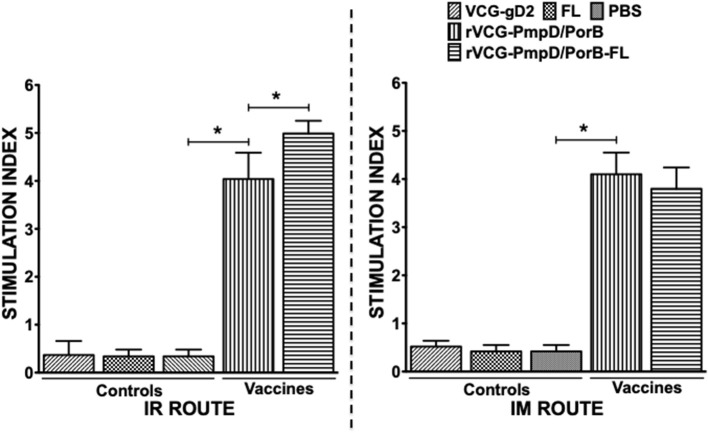
*Chlamydia*-specific CD4^+^ T cell proliferative responses. Four weeks after the last boost, splenic CD4+ T cells from groups of mice immunized via the IR and IM routes were restimulated *in vitro* with chlamydial antigen for 5 days. The antigen-specific proliferative response was determined using the 5-Bromo-2′-deoxy-uridine (BrdU) cell proliferation assay kit according to the manufacturer's instructions (Roche Molecular Biochemicals, Indianapolis, IN); BrdU incorporation was detected by addition of anti-BrdU antibody and the absorbance was read at 405 nm. Results are expressed as the stimulation index (SI), the ratio between absorbance values of stimulated and non-stimulated cells and the bars represent the mean and S.D. of six replicates from two independent experiments. Significant differences between experimental groups were evaluated at (*p < 0.05).

We compared the antigen-specific proliferative responses elicited following mucosal and systemic immunization by analyzing the stimulation indices (SI), the ratio of the absorbance values of antigen-stimulated and non-stimulated cells, resulting from stimulation of T cells purified from the spleens of immunized mice. The results show that irrespective of route of vaccine delivery, immune T cells from rVCG-PmpD/PorB-immunized mice proliferated significantly higher (*p* < 0.05) in response to antigenic restimulation compared to the vaccine carrier controls (PBS, rVCG-gD2, or FL) (Figure 3). SI values obtained following mucosal and systemic delivery of vaccine were comparable. Co-delivery of vaccine with FL resulted in significantly enhanced immune T cell proliferative responses following mucosal but not systemic vaccine delivery. Furthermore, the level of T cell proliferation induced by the adjuvanted vaccine was significantly higher (*p* < 0.05) following mucosal vs. systemic immunization. These results indicate that route of vaccine delivery influences the impact of FL on rVCG vaccine-induced cell-mediated immune responses.

### IR and IM Immunization Stimulated the Production of High Levels of *Chlamydia*-Specific Antibodies in Serum and Vaginal Lavage

**Figure 4:**
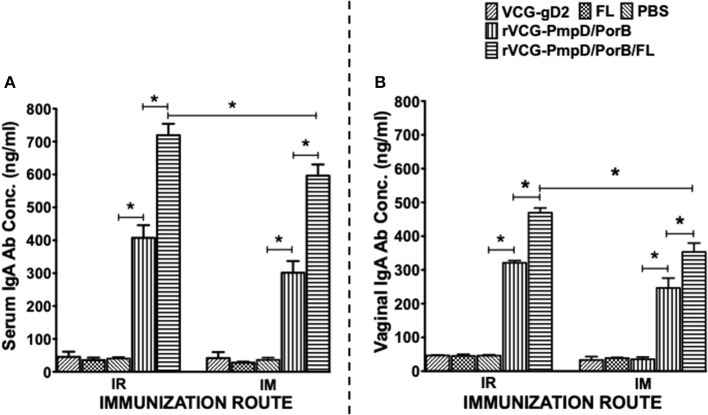
Chlamydial PorB-specific IgA antibody responses elicited after immunization. Groups of mice were immunized IR or IM three times, 2 weeks apart as described above. Serum and vaginal lavage samples were obtained at 2 weeks after the last immunization and pooled for each group. An antibody ELISA was used to assess the concentration of IgA antibodies elicited in serum (systemic) **(A)** and genital lavage (mucosal) **(B)** samples. Results generated simultaneously with a standard curve, display data sets corresponding to absorbance values as mean concentrations (ng/ml) ± SD of triplicate cultures for each experiment. The results are from two independent experiments with similar results and the data shown is from one of the experiments. Significant differences between experimental groups were evaluated at (*p < 0.05).

**Figure 5:**
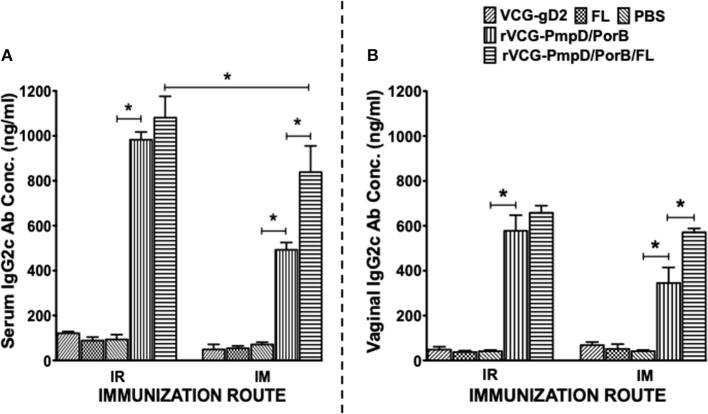
PorB-specific IgG2c antibody responses elicited after immunization. Groups of mice were immunized, and serum and vaginal lavage samples were collected and pooled for each group as described above. The concentration of antibodies elicited in the serum **(A)** and genital lavage **(B)** samples was assessed by a standard antibody ELISA assay. Results generated simultaneously with a standard curve, display data sets corresponding to absorbance values as mean concentrations (ng/ml) ± SD of triplicate cultures for each experiment. The results are from two independent experiments. Significant differences between experimental groups were evaluated at (*p < 0.05).

Two weeks after immunization, the concentration of antigen-specific antibodies elicited in the serum and vaginal lavage of vaccinated and control mice was measured by antibody ELISA. IR and IM immunization stimulated the production of PorB-specific IgA antibodies in both serum and vaginal lavage that were significantly higher (*p* < 0.05) compared to controls (PBS or rVCG-gD2 or FL (Figure 4). Furthermore, co-delivery of vaccine with FL significantly enhanced the magnitude of these responses in both serum (Figure 4A) and vaginal secretions (Figure 4B), irrespective of route of immunization. Similarly, mice immunized IR, and IM elicited significantly higher (*p* < 0.05) levels of PorB-specific IgG2c antibodies in serum and vaginal lavage compared to controls (Figure 5). The concentration of IgG2c antibodies elicited in serum and vaginal wash was significantly higher (*p* < 0.05) in IR immunized compared to IM immunized mice (Figure 5). Interestingly, in contrast with IgA, co-delivery of vaccine with FL significantly enhanced IgG2c antibody levels in serum (Figure 5A) and vaginal secretions (Figure 5B) after IM systemic but not IR mucosal immunization. The results indicate that route of vaccine delivery influences the impact of Flt3L on rVCG vaccine-induced humoral immune responses.

### Immune Effectors Elicited by Both IR and IM Immunization Protected Mice Against Challenge Infection With *C. muridarum*

**Figure 6:**
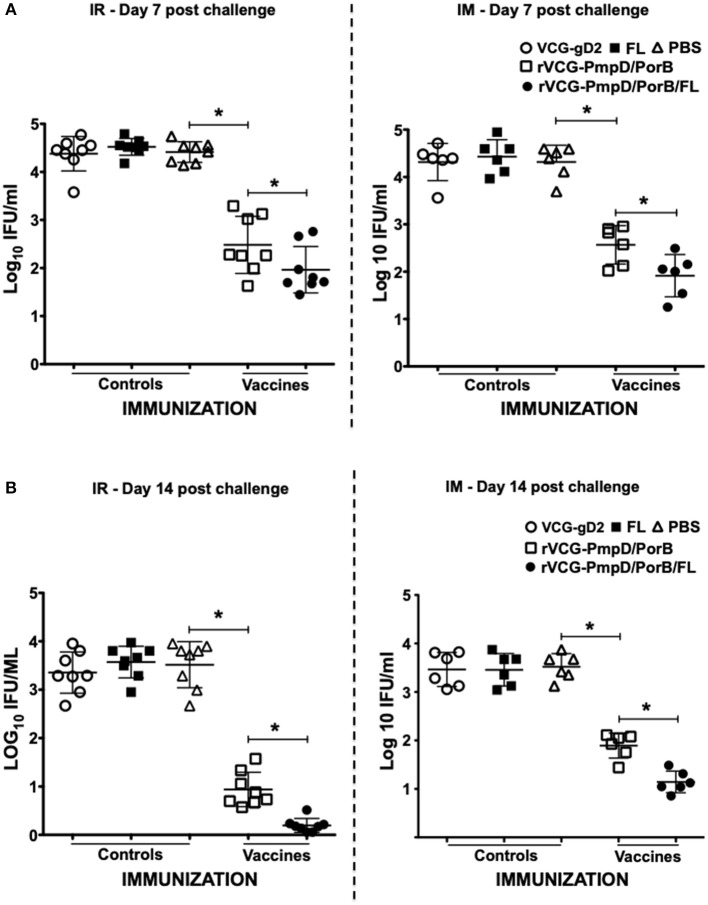
Cross protection against heterologous intravaginal challenge with *C. muridarum*. Groups of mice immunized IR and IM as described above were challenged intravaginally with 5 × 10^4^ IFU of live heterologous *C. muridarum* 2 weeks after the last immunization. One week prior to challenge, mice were administered Depo Provera to stabilize the estrous cycle and facilitate a productive infection. Infections were monitored by cervicovaginal swabbing of individual animals on days 7 and 14, and *Chlamydia* was isolated from swabs in tissue culture and enumerated. The data show the recoverable IFUs from each mouse expressed as log_10_ IFU/ml on day 7 **(A)** and day 14 **(B)** post-challenge. Differences between experimental groups were compared by Student's *t*-test at *p < 0.05. The experiment was designed to contain 6–8 mice per group.

To evaluate if elicited immune responses could protect mice against infection, immunized mice were infected with *C. muridarum* via the intravaginal route 2 weeks post-immunization and genital bacterial burden was assessed by isolation and enumeration of chlamydial IFU from cervicovaginal swabs at different time points. The results showed that by day 7 post-challenge, rVCG-PmpD/PorB-immunized mice shed fewer numbers of *Chlamydia* (up to 2-log lower IFUs) compared to the carrier (PBS, VCG or FL) controls, irrespective of route of administration (Figure 6A). By day 14 post-challenge, there was a further reduction in bacterial load in both IR and IM immunized mice (Figure 6B) with the IR-immunized mice shedding comparably lower bacterial loads. Moreover, co-delivery of vaccine with FL significantly (*p* < 0.05) enhanced bacterial clearance, irrespective of route of vaccine delivery (Figure 6). The results indicate that the immune effectors elicited by the mucosally delivered rVCG vaccine conferred superior protection compared to that elicited by systemic immunization.

### Adoptive Immunization With Immune CD4+ T Cells Protects Naïve Mice Against Live Chlamydial Challenge

**Figure 7:**
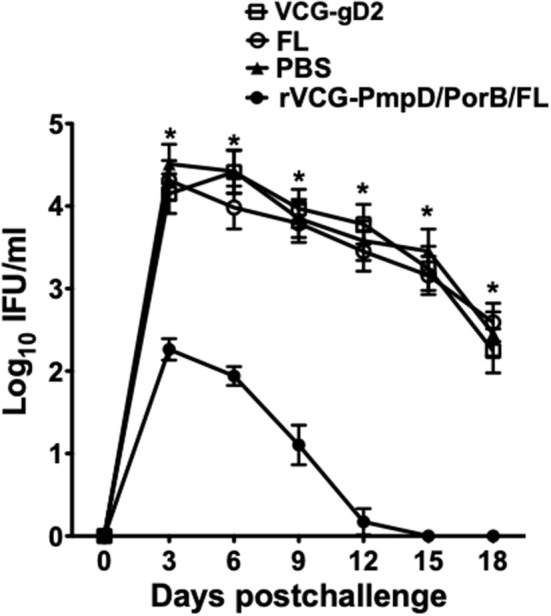
Protective efficacy conferred in naïve mice adoptively immunized with splenic CD4+ T cells from mice immunized with rVCG-PmpD/PorB/FL vaccine. Splenic CD4+T cells from mice immunized with rVCG-PmpD/PorB/FL or controls were adoptively transferred intraperitoneally into naive recipients at 2.5 × 10^7^ cells/mouse (2.5 spleen equivalents). The mice were then challenged intravaginally with 5 × 10^4^ IFU of live *C. muridarum* 24 h after cell transfer. The number of IFUs recovered from cervicovaginal swabs was determined every 3 days for 3 weeks post-challenge. The data show the mean recoverable IFUs, expressed as log10 IFU/ml, for each experimental group ± S.D. Differences between experimental groups were compared by Student's *t*-test at *p < 0.05.

To determine if immune CD4+ T cells could protect naïve mice against infection, CD4+ T cells purified from mice rectally immunized with rVCG-PmpD/PorB-FL were passively transferred intraperitoneally into naïve mice and then vaginally challenged with *C. muridarum*. Chlamydial burden was expressed on a log10 scale and shows the results of the vaginal shedding as mean IFU/ml (Figure 7). Significant protection was observed in the group of mice passively immunized with splenic immune CD4+ T cells as indicated by the decrease in chlamydial numbers shed and the length of time of chlamydial shedding compared to the controls (PBS, rVCG-gD2, and FL). In all, the group of mice passively immunized with CD4+ T cells isolated from the spleens of mice immunized with rVCG-PmpD/PorB-FL shed significantly less (*p* < 0.05) *Chlamydia* at all time points, when compared with the mice passively immunized with control CD4+ T cells. Notably, as early as day 6 post-challenge, splenic immune CD4+ T cell-immunized naïve mice shed about 2-log lower chlamydial IFUs compared to the group that received CD4+ T cells purified from control-immunized mice. Furthermore, 2 weeks after challenge, all the mice that had received the vaccine-derived CD4+ T cells were no more shedding *Chlamydia* compared with the mice passively immunized with control CD4+ T cells, which still shed high numbers of *Chlamydia* at this time. The results indicate that rVCG vaccine-derived CD4+ T cells play a significant role in protection against challenge with live *Chlamydia*.

## Discussion

We previously showed a rVCG-based multisubunit chlamydial vaccine (rVCG-PmpD/PorB) induced humoral and cellular immune responses in both systemic and mucosal tissues that significantly protected mice against intravaginal infection (17, 21). These results established the efficacy of the rVCG platform for inducing robust protective immunity even in the absence of external adjuvants. The route of vaccine delivery influences the nature of immune responses stimulated and the routing of immune effectors to the site of infection. Since both cellular and humoral immune responses contribute to *Chlamydia* immunity (34), a suitable vaccine delivery route should induce both mucosal and systemic immune responses. Mucosal immunization exploits the principles of the common mucosal immune system to direct immune effectors from one mucosal inductive site to other mucosal effector sites and is a practical method for vaccinating against mucosal pathogens. In this regard, IN immunization has been recognized as an effective mucosal route of vaccine delivery against genitally acquired microbial pathogens (35), such as *C. trachomatis*. However, the IN route may not be appropriate for delivery of all subunit vaccines, which often require co-administration with adjuvants as there are unmitigated concerns about the reactivity of some nasally administered adjuvants that may potentially cause neurological side effects in humans (36). The IR route was chosen in this study and will be preferred as an alternative mucosal route to overcome the challenges that may be imposed by IN immunization. Moreover, previous studies highlighted the potential of using intramuscular systemic and rectal mucosal routes for stimulating protective immune responses in the female genital tract (17, 21) and the incorporation of mucosal adjuvants in enhancing protective immunity (37). However, a direct comparison of rectal mucosal and intramuscular systemic routes of vaccine delivery and the effect of external adjuvants on chlamydial-specific immune responses following immunization with VCG-based vaccines have not been previously evaluated.

In the current study, we assessed the hypothesis that the impact of the FL on rVCG vaccine-induced chlamydial immunity is influenced by route of vaccine delivery. We first evaluated the impact of route of vaccine administration on chlamydial-specific immune responses. The results revealed that both routes of immunization induced the secretion by CD4+ T cells of comparably high levels of the Th1-associated IFN-γ and low levels of the Th2-associated IL-4 cytokines. These CD4 T cells also proliferated at comparable significant levels following mucosal and systemic delivery of vaccine. The induction of cellular immune responses characterized by chlamydial-specific CD4+ T cells that secreted high levels of IFN-γ following rectal immunization of mice with rVCG-PmpD/PorB have previously been reported (21). The results also showed high levels of IL-10 were secreted by immune T cells following both routes of immunization. *Chlamydia* species are able to modulate immune responses via regulating expression of some immune system molecules including cytokines. IL-10 is an anti-inflammatory cytokine which is produced by several types of immune cells including B and T regulatory and Th2 lymphocytes, activated macrophages and other cells (38). IL-10 is the most important regulatory factor suppressing the inflammatory functions of T and B lymphocytes as well as innate immune cells (39, 40). The secretion of high levels of IL-10 by immune T cells following both routes of immunization suggests that this cytokine may be playing a key role in the suppression of immune responses during immunization.

Further immunologic evaluation showed that both routes of vaccine administration stimulated the production of high levels of IgA and IgG2c antibodies in serum and vaginal secretions of immunized mice. Our finding of higher levels of the Th1-associated IgG2c compared to IgA antibodies in genital secretions confirm the results of our previous studies (21). These findings also agree with the results reported from human studies, which show that IgG rather than the secretory IgA isotype is the major immunoglobulin present in female genital tract secretions (41, 42). The association of the Th1-associated IgG2c antibody isotype with *Chlamydia* immunity has previously been reported (43) and support our suggestion that the antibodies that facilitate genital chlamydial clearance conceivably migrate from the systemic circulation rather than the genital tract (21). These results suggest that both mucosal and systemic routes of immunization efficiently facilitated the uptake and presentation of vaccine antigens to immune cells in mucosal and systemic immune inductive sites where strong immune responses were mounted against the vaccine antigens.

Targeting antigens to APC improves their ability to stimulate cellular and humoral immune responses. One such targeting strategy is co-delivery or co-expression of Flt3L with vaccine antigens (44). Previous studies have also established Flt3L as a suitable nasal mucosal adjuvant, augmenting protective immunity to a number of different antigens (29, 31). To date, the role of Flt3L as an adjuvant in vaccine-induced adaptive immunity against *C. trachomatis* has not been evaluated. Thus, we evaluated the efficacy of FL, which is known to stimulate the expansion of DCs, as an adjuvant to enhance both mucosal and systemic chlamydial immunity following mucosal and systemic delivery of vaccine. Notably, we found that co-delivery of vaccine with FL significantly enhanced Th1-type cytokine secretion and T cell proliferative responses after IR mucosal but not IM systemic immunization. Furthermore, co-delivery of vaccine with FL significantly enhanced the magnitude of IgA responses in both systemic and mucosal compartments, irrespective of route of immunization. Interestingly, in contrast with IgA, co-delivery of vaccine with FL significantly enhanced IgG2c antibody levels in serum and vaginal secretions after IM systemic but not IR mucosal immunization. While the reason for this discrepancy is unclear, the results indicate that route of vaccine delivery influences the impact of Flt3L on VCG vaccine-induced cellular and humoral immune responses.

To analyze the effectiveness of the vaccine, we first assessed if immunization with PBS or FL adjuvant alone or VCG alone had any non-specific anti-chlamydial effect. We then evaluated the ability of the chlamydial vaccine rVCG-PmpD/PorB with and without FL to protect mice against challenge with live *C. muridarum* based on reduction in genital chlamydial burden and length of chlamydial shedding. Vaccine efficacy analysis showed that the vaccine-induced immune effectors protected mice against live heterologous *C. muridarum* infection irrespective of route of vaccine administration, with the regimen incorporating FL having a protective advantage. The finding that mucosal and systemic immunization with PBS or FL or rVCG-gD2 did not reduce bacterial burden in the genital tract following challenge indicates that the protection observed in mice immunized with rVCG-PmpD/PorB vaccine was antigen specific. The mechanism of immune enhancement by FL appears to be due to its ability to increase the number of DC recruited to the immunization site to elicit robust antigen-specific cellular responses (45, 46). Moreover, another study demonstrated that co-expression of FL and GM-CSF considerably improved the ability of splenic DC to mature and present antigen to T cells, which was associated with an increase in the number of DC recruited to the immunization site, production of higher levels of IFN-γ and increased cytotoxicity of splenocytes in vaccinated mice (28). This study, which employed HER2/neu (a target antigen for anticancer therapy) as a DNA vaccine to study the adjuvant effect of murine Flt3L and GM-CSF in different forms of plasmid construction further demonstrated that FL targets DCs that constitutively express the receptor CD135.

The protective role of IFN-γ-secreting CD4+ T cells in both human and animal chlamydial immunity has previously been reported (7, 26, 47–50). Following the observation that rectal delivery of the FL-adjuvanted vaccine afforded better protection compared to intramuscular delivery, we asked if immune CD4+ T cells isolated from mice rectally immunized with the FL-adjuvanted vaccine could protect naive mice from intravaginal challenge infection. Thus, to confirm the role of CD4+ T cells in protection mediated by the VCG-based chlamydial vaccine, syngeneic naïve mice passively immunized with splenic CD4+ T cells purified from mice immunized IR with rVCG-PmpD/PorB-FL vaccine or controls were infected intravaginally with live *chlamydiae*. Importantly, CD4+ T cells from mock (PBS)- or VCG control-immunized mice failed to induce protective immunity against *C. muridarum* in naïve mice. The substantial reduction in genital chlamydial burden in mice passively immunized with splenic immune CD4+ T cells from rVCG-PmpD/PorB-FL vaccine-immunized mice confirms that the VCG-based chlamydial vaccine-derived CD4 T cells isolated from systemic tissues indeed have a protective function.

Taken together, the results strongly suggest that comparable protective immunity is elicited in the female genital tract against *Chlamydia* infection following mucosal and systemic immunization. The enhanced chlamydial clearance in the genital tracts of mice co-immunized with FL highlights the ability of FL to function as an effective immunostimulator at both mucosal and systemic sites. The higher magnitude of cellular and humoral immune responses, which also translated to better protection following IR administration of FL adjuvanted vaccine revealed that the immunomodulatory impact of FL on chlamydial-specific protective immune responses at mucosal and systemic sites is influenced by the route of vaccine administration. These differences may be related to differences in processing of antigens emanating from mucosal and systemic routes, differences in the quality of elicited immune effectors and capacity to seed immune effectors to distal mucosal and systemic tissues. Thus, direct targeting of VCG-based vaccines to antigen presenting cells by co-delivery with FL constitutes a viable immunization approach for inducing effective chlamydial immunity in the female genital tract.

## Ethics Statement

This study was carried out in strict accordance with the recommendations in the Guide for the Care and Use of Laboratory Animals of the National Institutes of Health. The Institutional Animal Care and Use Committee (IACUC) of Morehouse School of Medicine (MSM) (Assurance number A3381-01) approved the study protocol (Protocol Number: 16-15). Six-week-old female C57BL/6J mice (stock number 000664) (The Jackson Laboratory, Bar Harbor, ME) were used in this study. Mice were allowed to acclimate for 10 days in the MSM Center for Laboratory Animal Resources (CLAR) facility prior to experimentation. All immunizations, challenge and surgery were performed under ketamine/xylazine anesthesia, and conscious efforts were made to minimize suffering.

## Author Contributions

FE and RP conceived and designed the research. RP and YO performed the experiments. RP and FE performed data analysis and generated the graphs and figures. JI and KF participated in analyzing the data. FE wrote the paper. All authors have read and approved the manuscript.

### Conflict of Interest Statement

The authors declare that the research was conducted in the absence of any commercial or financial relationships that could be construed as a potential conflict of interest.
